# Integrated digital pathology at scale: A solution for clinical diagnostics and cancer research at a large academic medical center

**DOI:** 10.1093/jamia/ocab085

**Published:** 2021-07-14

**Authors:** Peter J Schüffler, Luke Geneslaw, D Vijay K Yarlagadda, Matthew G Hanna, Jennifer Samboy, Evangelos Stamelos, Chad Vanderbilt, John Philip, Marc-Henri Jean, Lorraine Corsale, Allyne Manzo, Neeraj H G Paramasivam, John S Ziegler, Jianjiong Gao, Juan C Perin, Young Suk Kim, Umeshkumar K Bhanot, Michael H A Roehrl, Orly Ardon, Sarah Chiang, Dilip D Giri, Carlie S Sigel, Lee K Tan, Melissa Murray, Christina Virgo, Christine England, Yukako Yagi, S Joseph Sirintrapun, David Klimstra, Meera Hameed, Victor E Reuter, Thomas J Fuchs

**Affiliations:** 1 Department of Pathology, Memorial Sloan Kettering Cancer Center, New York, New York, USA; 2 Institute of Pathology, Technical University of Munich, Munich, Germany; 3 Department of Health Informatics, Memorial Sloan Kettering Cancer Center, New York, New York, USA; 4 Department of Information Systems, Memorial Sloan Kettering Cancer Center, New York, New York, USA; 5 Department of Epidemiology and Biostatistics, Memorial Sloan Kettering Cancer Center, New York, New York, USA; 6 School of Medicine, Stanford University, Stanford, California, USA; 7 Human Oncology and Pathogenesis Program, Memorial Sloan Kettering Cancer Center, New York, New York, USA; 8 Department of Pathology, Icahn School of Medicine at Mount Sinai, New York, New York, USA

**Keywords:** digital pathology, whole slide imaging, computational pathology, artificial intelligence, honest broker, pathology

## Abstract

**Objective:**

Broad adoption of digital pathology (DP) is still lacking, and examples for DP connecting diagnostic, research, and educational use cases are missing. We blueprint a holistic DP solution at a large academic medical center ubiquitously integrated into clinical workflows; researchapplications including molecular, genetic, and tissue databases; and educational processes.

**Materials and Methods:**

We built a vendor-agnostic, integrated viewer for reviewing, annotating, sharing, and quality assurance of digital slides in a clinical or research context. It is the first homegrown viewer cleared by New York State provisional approval in 2020 for primary diagnosis and remote sign-out during the COVID-19 (coronavirus disease 2019) pandemic. We further introduce an interconnected Honest Broker for BioInformatics Technology (HoBBIT) to systematically compile and share large-scale DP research datasets including anonymized images, redacted pathology reports, and clinical data of patients with consent.

**Results:**

The solution has been operationally used over 3 years by 926 pathologists and researchers evaluating 288 903 digital slides. A total of 51% of these were reviewed within 1 month after scanning. Seamless integration of the viewer into 4 hospital systems clearly increases the adoption of DP. HoBBIT directly impacts the translation of knowledge in pathology into effective new health measures, including artificial intelligence–driven detection models for prostate cancer, basal cell carcinoma, and breast cancer metastases, developed and validated on thousands of cases.

**Conclusions:**

We highlight major challenges and lessons learned when going digital to provide orientation for other pathologists. Building interconnected solutions will not only increase adoption of DP, but also facilitate next-generation computational pathology at scale for enhanced cancer research.

## INTRODUCTION

Pathology is quickly transforming from an analog to a digital discipline.^1–3^ This includes scanning, reviewing, and storing of pathology slides in a digital format. In many studies, digital workflows have been reported to provide (1) faster turnaround time for retrieving digital cases without requiring manual administrative requests; (2) ability to review pathology slides at any time and any place where a computer is available, thus enabling remote work and telepathology; and (3) facilitated sharing of digital slides for consultations and educational purposes.^4–13^ The benefits of digital clinical workflows are currently evaluated individually per department,^14^ and groups have reported equivalency between digital and conventional pathology,^15–35^ and better efficiency after going digital.^36^^,^^37^

Recent advances in digital pathology (DP) further incorporate artificial intelligence and lead to new breakthrough technologies.^38–43^ Near-perfect models for pan-cancer diagnostic consensus,^44^ clinical-grade cancer detection,^45^ or novel molecular spatial biomarkers^46^^,^^47^ nicely example how next-generation DP has emerging impact in cancer research and patient care. However, developing these novel high-performing models requires large-scale, clinically relevant digital datasets. Such datasets can be found in diagnostic pathology, where tissue samples from cancer patients are routinely prepared, evaluated and archived, each associated with detailed pathology reports. Once digitized, these pathology data provide an extremely valuable treasure trove for computational pathology.

However, digitizing pathology slides at scale requires additional operational procedures on top of conventional diagnostic pathology. It makes large investments in whole slide scanners, redundant storage, and additional personnel necessary. The existing infrastructure needs to be adjusted to support a digital workflow (eg, digital slide viewers, workstations, laboratory information systems), and administrative and technical staff must be trained to use the new technology. For clinics, the digital slides must comply with the hospital’s quality standards, and they must be accessible by pathologists as easy and fast as possible, on-site and remote. For research, the compilation of large-scale datasets is complicated by the fact that image data, metadata, and patient consent information are distributed in different hospital databases that are not easily connected to each other, and that those data are not de-identified, impeding their export to research systems and collaborators. And for education, digital systems must be able to support anonymous access of didactic slides as well as concurrent access of digital slides by multiple trainees.

To meet these different requirements of clinicians, pathologists and researchers, a proper implementation of well-integrated, ubiquitously interconnected digital workflows in pathology is crucial. Earlier standard DP solutions are scoped for clinical or for research use only, do not integrate well into other hospital systems, and use proprietary data formats, programs, and workflows.

**Figure 1.**
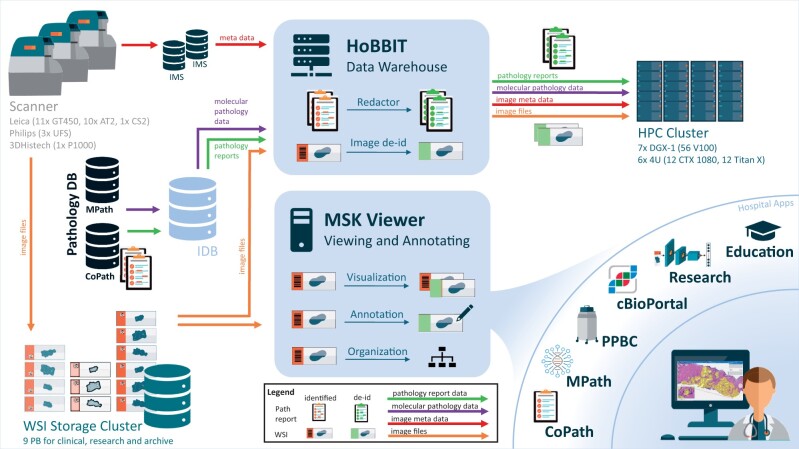
Overview of the digital and computational pathology landscape. Multiple scanner models (Leica AT2, Leica GT450, 3DHistech P1000, Philips UFS) digitize clinical and research slides and store them in our whole slide images (WSI) database. Metadata are sent to the vendor’s image management systems (IMS), forwarding them to the anatomic pathology laboratory information system (AP-LIS). The AP-LIS also hosts pathology reports and further clinical information. The MSK Viewer is connected to the WSI database and the IMS. Thus, it can visualize any digital image selected by image ID enabling integration into many hospital applications, such as CoPath, MPath, Precision Pathology Biobanking Center (PPBC), cBioPortal, research projects and education portal. For the clinical apps, authentication is leveraged with an authentication token, and protected health information (PHI) can be displayed. For research apps, PHI is hidden, and annotations can be gathered and shared across the systems. The Honest Broker for BioInformatics Technology (HoBBIT) combines data from digital slides with their meta and clinical information from the IMS, MPath, and CoPath together with patient consent information from the institutional database (IDB). It therewith provides large research datasets upon request by searching by clinical information, redacting pathology reports and de-identifying image data. Datasets are then stored on the high-performance computing (HPC) cluster, and a viewer project is optionally generated to visualize and/or annotate the images. DB: database.

To close this gap, we built a holistic solution for DP at the Memorial Sloan Kettering Cancer Center (MSKCC) over the last 3 years (**Figure 1**). Our digital workflow integrates in diagnostics, research, and educational systems using a single vendor-agnostic digital slide viewer that is cleared by New York State provisional approval for remote sign out during the coronavirus disease 2019 (COVID-19) pandemic. Images and annotations across the systems can be shared with or without protected health information (PHI). Our solution further includes a service linking clinical pathology data with research, thus enabling to quickly compile large-scale datasets for computational pathology including anonymized images, redacted pathology reports and clinical data of patients with consent. We review the system; show multiple use cases for clinical pathology, research, and education; and elaborate important landmarks and lessons learned.

### Background and significance

In contrast to other studies that focus on clinical use cases only, we elaborate in addition on the integration of the platform into research and education. For example, we show how the system has been used to develop novel computational models with clinical impact including the development of clinical-grade cancer detection for prostate needle biopsies, basal cell carcinoma, and breast cancer metastases. We illustrate the importance of a well-integrated DP in clinics, research, and education to facilitate and drive the adoption of the digital transformation. Further, no study exists describing a systematic workflow to generate large-scale datasets for research. We provide new insights into a DP ecosystem at a tertiary academic center, highlighting key aspects and metrics to consider when going digital in a heterogenous scanner landscape.

## MATERIALS AND METHODS

### Scanning landscape

The scanning operation at MSKCC is divided into routine anatomic pathology and archival scanning. Routine pathology scanning is further differentiated between prospective and retrospective scanning for pre- and postpathologist review and sign-out, accordingly. All prospective slides get scanned, while only a subset of designated retrospective slides is marked for scanning during the pathologist review.

Our device landscape grew over time. Current high-throughput scanners include 9 Aperio AT2s (Leica Biosystems, Buffalo Grove, IL) that are used for retrospective scanning for surgical, hematopathology, cytology, and molecular slides including hematoxylin and eosin (H&E), immunohistochemistry (IHC), and frozen section slides; 3 Aperio GT450 that are used for prospective H&E and IHC scans; an additional Aperio CS2 for backup manual scanning; 3 IntelliSite UltraFastScanner (Philips, Eindhoven, the Netherlands) for prospective H&E and IHC slides; and 1 Pannoramic 1000 (3DHistech, Budapest, Hungary) for whole mount and cytology slide scanning for both internally generated and external consultation slides. Internal slides are affixed with a barcode label generated in the anatomic pathology laboratory information system (AP-LIS) upon accessioning or at the time of IHC ordering. External consultation slides are affixed with a secondary MSK barcode label upon accessioning while preserving the referring institution’s original slide label. Certain whole slide scanners are able to automate decoding the MSK specific barcode on the additional MSK slide label, even with multiple (internal and external 2-dimensional barcodes), others are manually covered with ink or stickers as described earlier.^48^ The barcodes are read by the scanners in order to automatically assign the digital whole slide images (WSI) to the correct cases in the AP-LIS. For archival scanning, 1 Aperio AT2 and 8 Aperio GT450 are utilized at an off-site archival storage center to digitize all archival slides with machine readable barcodes on the slide labels. Slides older than 2012 do not have such barcodes and are currently not being scanned.

#### Quality control

Most scanners provide internal quality control procedures to ensure a high scan quality, but these systems are not sufficient. Incorrect focus points, scanning and glass slide artifacts or missing tissue (Supplementary Figure S1) are not always detected by the different scanners. Therefore, we employ a manual postprocessing after scanning. First, the macro image of each WSI is verified by a technician for obvious artifacts. Second, WSI from the Leica scanners with a scanner issued “Quality Factor” of <90 are then reviewed in detail to check the focus. Finally, every WSI from a Philips scanner and every 10th slide from the Leica and 3DHistech scanners is manually opened to verify successful scanning of the whole tissue without artifacts. In addition to this manual workflow, the viewer shows a macro image for every slide and enables pathologists to easily report poor quality slides or missed tissue to the scanning group, such that those slides can be quickly rescanned (Supplementary Figure S2).

### Storage

**Figure 2.**
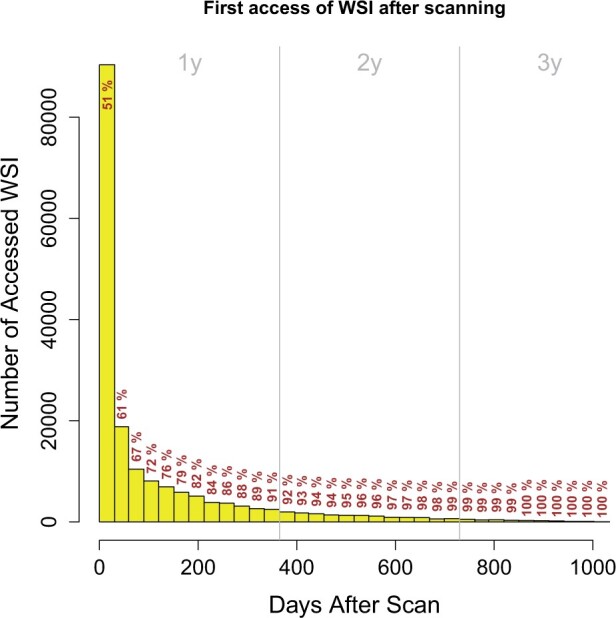
Histogram of the digitally reviewed whole slide images (WSI) ordered by their access time after scanning. Each bar contains the number of WSI first accessed in the corresponding month after scanning of the slides. Note that this plot only includes digitally reviewed WSI, and not all scanned WSI. Gray vertical lines indicate years. Cumulative percentages of accessed WSI are given. As a reading example, 51% of the digitally reviewed WSI were accessed within 1 month after scanning, 61% within 2 months, and 91% within 12 months.

WSI are hosted on a centralized data share in the hospital’s data center. Assuming an average size of 1 GB/WSI and a yearly scanning rate of 1 million scans, we estimate a need of 1 PB/year of secure and redundant storage. To meet that requirement for the next 6 years, we allocated a total of 9 PB for slide storage (3 PB of which are already used). To reduce storage cost, a 2-tier storage cluster has been employed keeping the equivalent number of slides for 6 months scanning on a tier-1 flash storage for fast access in the viewer. Older slides are automatically stored on less expensive tier-2 disk storage. The threshold of 6 months was estimated based on the usage of the slide viewer for 3 years in our hospital (**Figure 2**): From all WSI that have been reviewed digitally, 79% were opened within 6 months after scanning. We used this threshold as an estimate for the total amount of tier-1 storage needed for all scanned slides.

### Slide viewer

**Figure 3.**
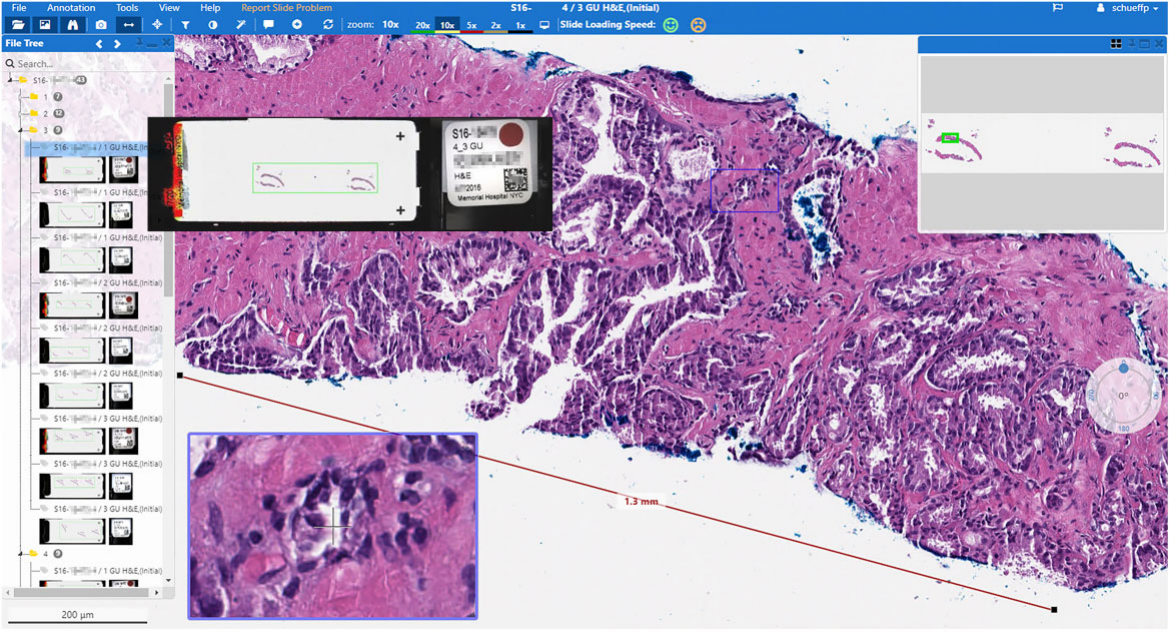
Screenshot of the MSK Viewer in a clinical context. Shown is a prostate needle biopsy with a measurement of the cancer extend. On the left side, the parts and slides of the case are listed together with the macro and label image for the pathologist to verify the scanned tissue and correct slide to be shown (enlarged on mouseover, protected health information blinded for publication). The viewer provides standard tools such as magnifier, overview image, and measurement tools, and can be opened on any computer or laptop within the hospital’s network.

To visualize, annotate and organize the WSI, we developed a vendor-agnostic web-based universal slide viewer (**Figure 3**, see Supplementary Appendix for technical implementation details). This customized solution opens WSI from the hospital’s AP-LIS (Cerner CoPathPlus), from a molecular pathology platform (MPath), a genomic research engine (cBioPortal), a pathology biobank (Precision Pathology Biobanking Center [PPBC]), and dedicated research projects.

To integrate with the different hospital applications, we implemented a generic application programming interface (API) to open a case or a particular image ID, using hospital-wide standard identifiers (**Table 1**). This API can be reused in many scenarios and easily adapted to connect to other applications.

**Table 1.** Important identifiers for a digital pathology platform

Identifier	Description	Example
Medical Record Number	Healthcare organization–specific identifier given to each patient.	1482928
Accession Number	Identifier for an accessioned case. Starts with a letter (S [surgical], C [cytology], H [hematopathology], R [research], M [molecular]), followed by the year, a dash and a running number. A patient can have 1 or multiple accessions.	S20-0123
Part ID	Organs are virtually divided into different parts to identify locations of specimens in the organ.	2
Block ID	Tissue of specific parts can be fixed in 1 or more paraffin blocks. The block ID identifies the block a specimen originates from.	3
Slide ID/Barcode	The unique ID of a glass slide with a prepared tissue specimen. The barcode is provisioned by the barcoding system/AP-LIS and a barcode sticker is pinned on the slide.	S20-0123; S12NSKJ
Image ID	The unique ID of a digital image. Provisioned by the scanner’s image management system. Can be a number, GUID or of different format. A physical slide can have multiple digital images, eg, when a slide is rescanned. Different scanner vendors use different ID schemes to avoid cross-vendor overlap of IDs.	936DA01F-9ABD-4D9D-80C7-02AF85C822A8
Image File Path	The physical path to the digital image file on the data share. Usually maintained by the IMS.	\\path\to\file.svs

These identifiers can be used to communicate across systems, and to search and open image data. All IDs except part and block number are considered PHI or sensitive and need to be redacted for external sharing.

AP-LIS: anatomic pathology laboratory information system; GUID: globally unique identifier; PHI: protected health information.

To facilitate authentication and to allow pathologists to access PHI in the viewer only when launching it from a clinical application such as the AP-LIS or MPath, the system leverages the hospital’s active directory (LDAP [lightweight directory access protocol]) for single sign-on. Connected clinical applications using LDAP can thus authenticate the user to see PHI, while research applications or nonauthenticated access will hide PHI to protect the patients’ confidentiality (Supplementary Figure S3). Standard viewers do not provide this flexibility: once you open a WSI, you will have access to its whole content including PHI, regardless of the semantic context. This imposes a privacy concern in cases in which one wants to share a date for research or education.

### Computational pathology research from consented patient data

To quickly compile and share large-scale research datasets in pathology for MSK internal and external use, we developed an Honest Broker for BioInformatics Technology (HoBBIT) (see Supplementary Figure S4 for architecture diagram) as part of our DP ecosystem. This service is connected to the AP-LIS, the institutional database, the scanners’ image management systems, the WSI storage, and the viewer. HoBBIT centralizes a data warehouse of DP with associated reports and clinical data, allowing us to easily search, filter, and compile relevant digital datasets at scale. HoBBIT takes information of patient signatures of Notice of Privacy Practice, demographic information and synoptic consent of enrollment in research protocols into account to ensure that records are properly included from research studies based on patient-level inclusion criteria. Each research project using HoBBIT requires institutional review board approval. After approval, requested compiled datasets are de-identified in accordance with the Safe Harbor method of the Health Insurance Portability and Accountability Act (HIPAA) by applying *date truncation*, *ID generation*, and *text redaction* on applicable data fields.

For *date truncation*, any dates discretely stored in the HoBBIT database are truncated removing the day and month, leaving the year.

For *ID generation*, novel de-identified HoBBIT identifiers (HIDs) are created to correspond to direct or indirect unique identifiers in the source data. The keys between the original and the de-identified identifiers are maintained in HoBBIT. We use this method to mirror 3 identifiers in the source data: (1) patient HIDs for Medical Record Numbers, (2) case HIDs for pathology accession numbers, and (3) image HIDs for image IDs from the vendor systems.

For *text redaction* to create de-identified versions of pathology reports, any identifiers located in the reports are replaced with placeholder text. This process is achieved by a combination of the vendor software De-Id^49^ with a homegrown algorithm that uses regular expressions to locate and redact dates, accession numbers, and medical record numbers from reports. The algorithm also searches for the true name of the patient and replaces it with placeholder text.

#### Image de-identification

The HoBBIT database stores image IDs and pointers to the corresponding file locations of the WSI. For requested datasets, the corresponding WSI are copied to the requestor. Still, originating from clinical cases, the copied WSI contain PHI and must be de-identified before being used for research to protect patient privacy. PHI can be found in 4 parts of a WSI: (1) in an overview image of the slide’s label sticker containing barcode and patient name; (2) in an overview of the whole glass slide (macro image)—the macro image can contain sensible parts of the label sticker; (3) in text metadata such as barcode, accession ID, scanning dates, and other sensible information; and (4) in the main high-resolution image when sensible parts of the label sticker have been scanned (eg, when the sticker was put close to the tissue).

To de-identify a WSI according to points 1 to 3, the label image, the macro image and sensible metadata are removed by overwriting the corresponding data. As the file formats vary across vendors, this process must be developed separately per vendor. For Leica scans, we employ an opensource script^50^ to remove PHI and sensitive information from the WSI. For Philips scans, we employ a home-grown de-identification script. For 3DHistech scans, we employ the graphical user interface–based SlideConverter feature that is included in the CaseCenter software.

To exclude PHI in the main high-resolution image data as stated in point 4, we incorporate an automated detection step for dark image areas as they arise from scanned label stickers on the digital thumbnail image. If such areas are detected, the corresponding WSI is excluded from the dataset to virtually eliminate the risk of PHI exposure.

#### Computing

HoBBIT can share the de-identified WSI and pathology reports internally or externally. For internal use, WSI are optionally transferred to a computing cluster and/or added to a viewer project to enable gathering of additional image annotations and to facilitate review of data and model outputs. The high-performance computing cluster comprises 7 NVIDIA DGX-1 nodes with 8 Tesla V100 GPUs each, as well as 6 servers with 12 GTX 1080Ti GPUs and 12 Titan X GPUs and 512 GB RAM, each. It further includes 5 PB of GPFS (Global Parallel File System) high-performance storage for raw data. All systems are connected by a 100 Gb/s network backbone internally, and with 1 to 10 Gb/s connections externally to peripheral hospital systems. This specification enables training of deep neural network models on previously compiled massive image data.

#### Education portal

With the use of HoBBIT de-identification engine and an institutional licensed Web-based software, PathPresenter, trainees and pathologists can directly migrate educational digital slides or cases to the MSK Pathology Education Portal. An API was developed to anonymize digital slides, and send diagnostic or relevant information with each slide to the portal. The platform allows for searching of digital slides by diagnosis from aggregated slides migrated by pathologists or trainees. Interactive cases can be created with digital slides and dynamic annotated content for users to review.

## RESULTS

**Figure 4.**
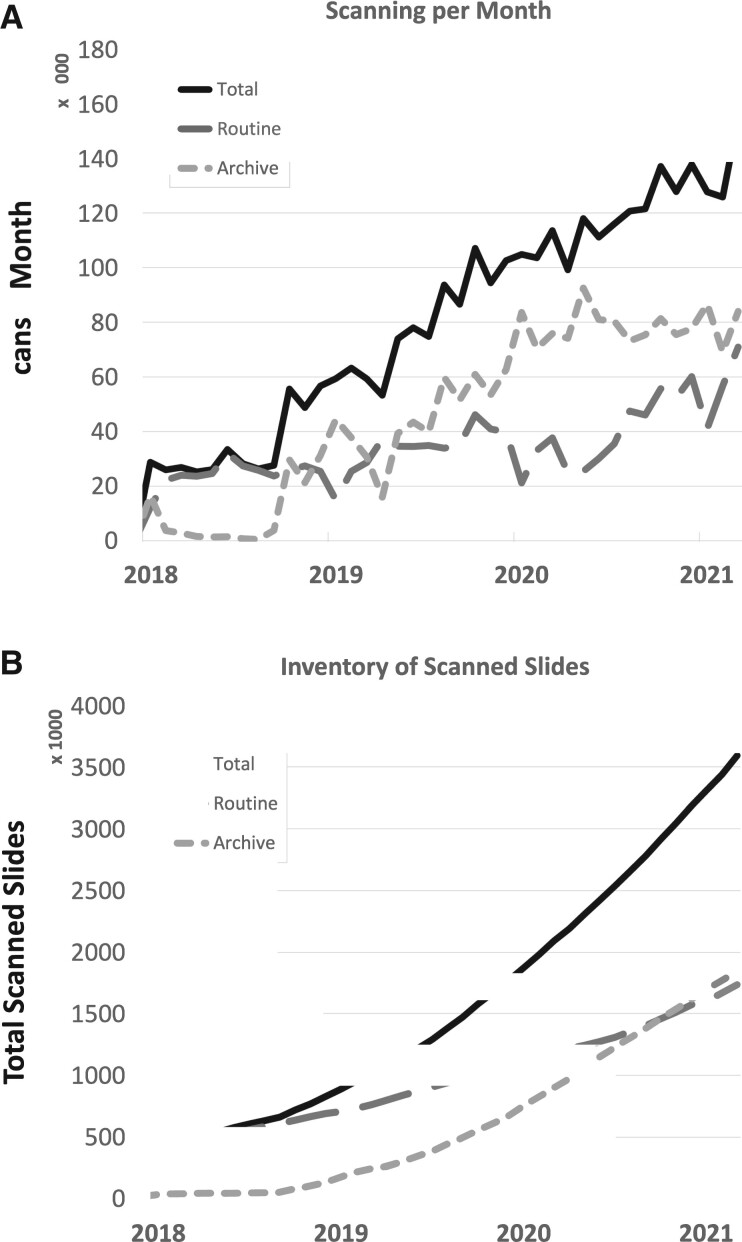
Scanning effort at Memorial Sloan Kettering Cancer Center over time. (A) The overall inventory contains 3.6 million digital slides and continuesto grow. (B) Currently, 140 000 slides are digitized every month for both routine diagnostic and archival scanning.

We summarize key results of the DP ecosystem evaluated over the last 3 years. As of March 2021, 3.6 million glass slides have been digitized at a total scanning rate of 140 000 slides/month including 1.8 million slides from each the routine anatomic pathology and the slide archive (**Figure 4**). The file size of WSI ranges from 9 MB to 40 GB, varying with scanner model, magnification and resolution of the scan,^51^ and the amount of tissue. As compared in **Table 2**, scans with a spatial resolution of 0.5 µm/pixel (Aperio AT2) have a median file size of 403 MB, and the file size increases with the resolution to a median of 1105 MB (Aperio GT450, 0.25 µm/pixel), 1418 MB (UFS, 0.25 µm/pixel), and 5752 MB (P1000, 0.12 µm/pixel). Although all scans have been processed by our quality control (QC) pipeline, pathologists reported 31 scans for blurriness, 7 for missing tissue, 5 for air bubbles, and 7 for other reasons using the viewer in 2018 and 2019, and those slides were rescanned within a day.

**Table 2.** Average sizes in MB of scans from different scanner models at Memorial Sloan Kettering Cancer Center

		Aperio AT2	Aperio GT450	Philips UFS	3DHistech P1000
Slides		n = 2142	n = 2260	n = 2290	n = 1600
Magnification		20x	40x	40x	40x
Resolution		∼0.5 mpp	∼0.26 mpp	0.25 mpp	∼0.12 mpp
Compression		JPEG and J2K (Q = 80)	JPEG (Q = 91)	Proprietary	JPEG (Q = 80)
File format		SVS	SVS	iSyntax	MRXS
File Size (MB)	Mean (SD)	489 (377)	1171 (566)	986 (739)	5878 (3155)
	Minimum	9	92	40	75
	Q1	182	753	392	4347
	Median	403	1105	727	5491
	Q3	711	1509	1463	6978
	Maximum	2660	4168	5002	27 676

Samples have been randomly selected from 1 day (Aperio) or multiple days (Philips, 3DHistech). File sizes approximately increase by the factor of 4 when doubling resolution. We scan with a mixture of 20× and 40×.

mpp: microns per pixel.

### Clinical integration for facilitated sign-out

To review WSI in routine pathology and remote sign-out sessions, we utilize a vendor-agnostic Web-based digital slide viewer. During its development, 2 feedback studies with 8 and 12 pathologists, respectively, have been conducted to assess specific requirements and features (see Supplementary Appendix for study description details). As listed in **Table 3**, the most prominent feature request was the ability to review 1 or multiple WSI from within specific hospital applications (CoPath AP-LIS, MPath, cBioPortal, and PPBC), regardless of the scanner make and model within a single viewer. Note that the conventional behavior of the AP-LIS is to open different viewers for different vendor’s interfaces, including cases with mixed WSI originating from different scanner vendors. This increases complexity for the pathologist due to multiple windows on the screen. Our solution connects to all AP-LIS interfaces, and WSI from different vendors are opened in the same viewer, providing a single loo-and-feel and reducing complexity to the pathologist.

**Table 3.** Required features by our pathologists for a viewing system in our workflows, including features that are not standard in digital slide viewers

Commonly required features for a viewing system by pathologists		
1.	Integration	Be able to open WSI from different scanner vendors in one application, without installing additional software, on different operating systems (Windows, MacOS)
2.		Be able to open WSI from clinical systems (such as Cerner CoPathPlus and MPath) with 1 click, using single-sign-on, authenticated to see PHI such as label and macro images
3.		Be able to open WSI from research systems (eg, cBioPortal and PPBC) with one click, using single sign on and without exposing PHI
4.		Be able to send individual slides with annotation easily to an education portal, without exposing PHI
5.	Share and download	Be able to download images in an anonymized format for educational purposes
6.		Be able to easily share anonymized WSI and annotations with trainees
7.		Be able to easily share WSI among colleagues for consultations
8.	Annotate	Be able to assemble slide collections for education and research projects
9.		Be able to pixelwise annotate images and share annotations across research groups
10.	Efficiency and automation	Be efficient and do not lose performance (speed, image appearance)
11.		Be supported by automation tools such as measurement tools, screenshot tools, predefined mitotic count boxes, and others
12.		Be able to recognize reviewed slides and optionally reviewed areas on slides
13.		Be able to flip and rotate images and annotations
14.		Be able to review multiple slides side by side
15.		Be able to quickly detect poor quality scans and send them to rescan
16.	Ergonomics	Be able to efficiently navigate and annotate images with different input devices than a mouse

These features have been assessed in 2 feedback studies.

PHI: protected health information; PPBC: Precision Pathology Biobanking Center; WSI: whole slide images.

**Figure 5.**
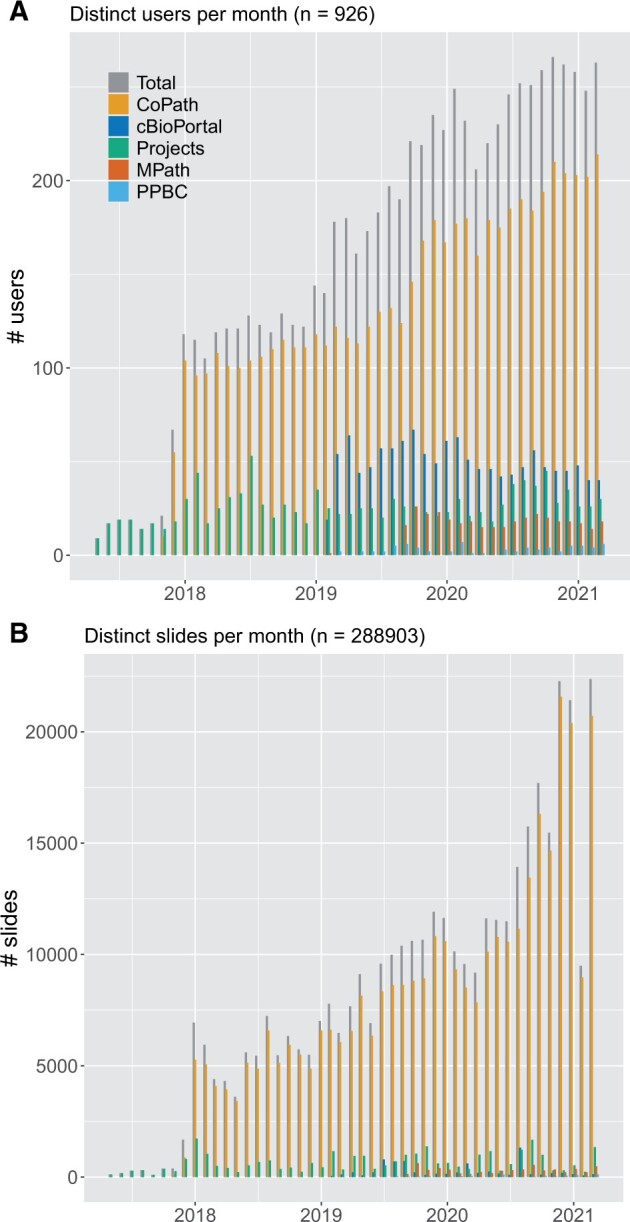
Number of (A) distinct users and (B) distinct slides per month processed by the digital slide viewer. Our platform launched 2017 as a research tool for annotations of individual slides in specific research projects. Starting 2018, it became the main viewer for clinical cases in the anatomic pathology laboratory information system. In 2019, more systems have been connected to the MSK Viewer, including cBioPortal and MPath. Each of those systems increased the number of users steadily, as pathologists and researchers use the viewer for different tasks. After approval of the digital workflow from New York State regulatory bodies in mid 2020, significantly more slides have been accessed via CoPath. In total over the last 3 academic years, 926 users (from CoPath: 455, cBioPortal: 449, Projects: 291, MPath: 50, and Precision Pathology Biobanking Center [PPBC]: 18) accessed 288 903 whole slide images in the viewer.

The AP-LIS workflow is used by surgical pathologists, molecular pathologists, fellows, trainees, rotators, laboratory and scanning personnel for reviewing prior cases, IHC, special stains, and consultations. Currently, we count over 175 distinct users per month (418 total over the last 3 years) originating from CoPath alone (**Figure 5**). The MSK Viewer is utilized daily for clinical review of prospective and retrospectively digitized whole slide images. The retrospective review from patient’s prior material has transformed the Department of Pathology, in which 97% of routine glass slide requests have been obviated by the direct access of the digital slides.^48^ This has been specifically useful for review of patient’s prior pathology at the time of frozen section or during review of patient follow-up surgical excisions, and for tumor morphology comparisons between locally recurrent or metastatic disease.

For diagnostic molecular pathology, MSKCC employs its own, customized software suite known as MPath that includes numerous case processing and next-generation sequencing analysis tools. It associates molecular cases in its own laboratory information management system with surgical cases and opens the corresponding images via links in the pertinent application. This enables efficient and easy review of molecular results alongside with case histology. The ease with which WSI can be accessed allows teams to review the WSI during group sign-out while the details of the molecular results are being discussed, allowing for education on molecular-morphological correlations and review of slides for quality-related issues (eg, low tumor content or possibility of contaminant material). Before integration, cases had manually to be opened in the AP-LIS, requiring multiple programs to be opened and locking the case during molecular assessment.

### Equivalency for digital sign-out and remote digital pathology

In order to use these digital workflows for primary diagnosis in New York State, where the New York State Department of Health acts as the CLIA-deemed entity for the Centers for Medicare and Medicaid Services, a laboratory developed test was pursued. This validation and submission has been published,^52^ and included a large prospective digital pathology concordance study including remote review of patient pathology.^53^ The result was a successful validation study with 100% major diagnostic concordance, including top-line diagnosis, margin status, lymphovascular and/or perineural invasion, pathology stage, and the need to order ancillary testing. These results were submitted to the New York State Department of Health, which received a provisional approval for use of the described clinical digital workflows for primary diagnosis, including in the remote setting as it is used during the public health emergency in the COVID-19 pandemic.

### Integration into research applications cBioPortal and PPBC

The Web-based viewer allows for the accessing of 1 or multiple WSI via API. As a result, integration into hospital applications is easy and generic. For example, we connected the viewer with cBioPortal, a platform for the visualization and analysis of large-scale cancer genomics datasets.^54^^,^^55^ Amongst others, it houses the genetic information of the IMPACT tumor profiling program,^56^ in which patients are routinely tested for mutations in 468 genes to improve molecular classification, treatment, and cancer research. Upon integration, genomic cases in cBioPortal exhibit a new additional tab in the system containing the viewer for the corresponding WSI (Supplementary Figure S3). The WSI are identified by their image IDs stored in the cBioPortal database. The API of the viewer allows for the visualization of the correct and de-identified images via URL in an iFrame in cBioPortal. This connection between genetic and image information in a centralized platform is an important step to a more complete understanding of cancer and facilitates research in a collaborative setting between computational biology and pathology.

A second connected research platform is the PPBC. This is a central hub hosting tissue-based research samples from over 88 000 unique patients including 38 000 histology slides, 4000 H&E- and 7000 IHC-stained slides for clinical trials, new diagnostic assay development, and biospecimen research. Histopathologic evaluation of tumor and matched normal tissue is a frequent prerequisite for this research, but pathology review using glass slides from off-site storage poses a bottleneck that impacts laboratory turnarounds. Often, the same material is of interest to multiple investigators spread across the institute thereby making glass slides inaccessible for the research pathologists. The integration of the digital platform to PPBC has enabled core pathologists to perform pathology review of WSI via a link that associates the specimen with corresponding digital images. When clicking on the link, the WSI are securely opened via API in the preferred Web browser, thereby eliminating the need for glass slides altogether. As a result, the requisitions for glass slides have decreased by over 70%.

Both research integrations are less frequently used than the clinical one (cBioPortal: 50 users/month, PPBC: <10 users/month) (see **Figure 5**). Still, both have shown to improve the respective workflows by providing a more native experience to the user (eg, WSI in cBioPortal) and reducing the need of ordering physical glass slides (eg, PPBC). The flexibility of our system allows for the easy integration into additional systems in future as well with the potential to enrich other research databases with digital pathology image data.

### Integration into stand-alone research studies

The viewer further allows for the organization, visualization, and annotation of WSI in various collaborative research projects. Such a project is a collection of digital image IDs, organized in a flexible folder structure. Each user can create new projects as soon as the image IDs are known. Image IDs can be gathered via HoBBIT or manually using case IDs via the IMS. In the viewer, the project’s WSI can be annotated with points, rectangles, polygons, arrows, and text annotations, as well as with pixel-accurate, freehand drawings using a mouse or a pen. Individual label schemes can be defined per project that include different colors or classes.

Projects are private but can be shared among collaborators and supervised by other pathologists and researchers, according to roles and permissions. A supervision role includes review, correction, and approval of annotations and is helpful for projects with multiple annotators, or for a senior pathologist to approve annotations of junior fellows.

The presented workflow using HoBBIT to systematically filter and compile pathology datasets and using the viewer to review and annotate WSI and evaluate and discuss model outputs has been used in numerous peer-reviewed published research studies for the development of advanced computational models, including clinical-grade cancer detection models for prostate biopsies,^45^ deep interactive learning for efficient WSI labeling,^57^ saliency annotation and prediction for pathologist at the microscope,^58^ cancer subtyping approaches,^59^^,^^60^ evaluation of frozen section accuracy after neoadjuvant chemotherapy for breast carcinoma,^61^ quality control and pen annotation extraction of WSI,^62^^,^^63^ cell nucleus detection,^64^ and deep multimagnification networks for breast cancer segmentation.^65^

### Integration into an educational system

Besides routine anatomic pathology and research, the viewer adds to the institutional education program in 2 specific ways. First, de-identified versions of didactic pathology slides can be downloaded by students and saved for educational use. This opportunity is highly utilized: over the 2019 academic year, 2135 WSI were downloaded using this workflow (Supplementary Figure S5). Second, we included the option in the viewer to send single WSI with a comment via a context menu to a dedicated education portal using PathPresenter centralizing select images for educational access by professional staff and trainees. The portal was initiated in 2020 and supports 153 active users. Users have sent 7211 digital slides with diagnostic metadata from the MSK Viewer to the portal. Trainees and pathologists have access to their own virtual slide collections from the Web-based platform as well as publicly available crowd-sourced pathology digital slides. The portal also provides other tools such as integrating digital slides into presentations, conferencing workflows, and interactive case creation with interactive annotations. These case groups have been used to create a frozen section challenging cases module to enable pathologists the ability to review anonymized discordant frozen section interpretations with paired frozen section slides and control sections with anonymized clinical metadata. Other education modules that have been developed include a genitourinary pathology and breast pathology high-yield case modules, currently with over 50 unique annotated cases.

There is an unspecific advantage for education with our solution as well: namely, the possibility for students to launch anytime and anywhere educational cases from the AP-LIS in the viewer without the need to order the slides physically, thus allowing simultaneous and remote access. Additionally, the education portal hosts anonymized images and can be accessed on any device with an internet connection. This enables pathologists and trainees the ability to review digital slides anywhere. To the best of our knowledge, this is the first integration of such a programming interface to support de-identified clinical metadata and corresponding anonymized digital slides to enable educational content.

The integration to the many hospital applications is important for adoption and interconnectivity, but also specific to a custom DP solution. Standard viewers are bound to their vendor’s systems such that many viewers would be needed to interact with the different hospital applications (Supplementary **Figure S6**). A universal viewer, however, enables full integration at the hospital and provides a uniform look and feel for the user. **Figure 5** illustrates the relevance of these integrations for a broad acceptance of the DP workflow: at initial launch in 2018, the viewer connected to the AP-LIS alone, with 100 distinct users per month. In 2019 and 2020, MPath, cBioPortal, and PPBC have been connected to the viewer, steadily increasing the footprint to 225 distinct users per month. In total over the last 3 years, 926 users (from CoPath: 449, cBioPortal: 455, Projects: 291, MPath: 50, and PPBC: 18) accessed 288 903 WSI in the viewer.

## DISCUSSION

We introduced a ubiquitously integrated DP ecosystem installed and validated over 3 years at the MSKCC. A vendor-agnostic universal digital slide viewing and annotation platform integrates into the AP-LIS, the clinical molecular pathology platform MPath, the open genomics research database cBioPortal, and the tissue biobank PPBC. Further connections to more systems are planned and easily feasible due to a generic Web API. This comprehensive integration of the viewer is crucial for the adoption of DP by pathologists as it provides a single look and feel and facilitates annotating and sharing. The connection to the AP-LIS alone is not sufficient, as pathologists use additional systems in their workflows. No solution existed before that provides the flexibility to connect to the whole application landscape. The system can be used not only in clinical workflows displaying required PHI to the pathologist, but also in a research or educational context hiding PHI to protect patient’s privacy.

Our current setup supports over 200 distinct users accessing 10 000 images per month. It has been extensively validated in 2 reader studies, and it is the first home-grown DP system cleared by New York State provisional approval in 2020 for primary diagnosis and remote sign-out. This enables pathologists to review WSI during the current COVID-19 pandemic in a remote setting, which is an important aspect for the hospital.

To improve the digital workflow, work is planned to order additional stains from within the viewer, add clinical consultation queues for sending or receiving digital cases for a second opinion, and push diagnostic reports and annotations directly from the viewer back to the AP-LIS.

We also outline a solution to easily compile clinically relevant pathology datasets at scale. This enables us to use the massive digital slide archive of the hospital for cancer research. To this end, a central data warehouse connects to the AP-LIS, institutional database, and slide scanner systems to correlate all necessary data such as WSI, pathology reports, clinical data, patient consent and institutional review board approvals. All data are de-identified and redacted prior their use for research. As scanner vendors get more and more aware of the dual use of WSI, they can provide proper de-identification software for their proprietary file formats.

## CONCLUSION

The increasing excitement for DP and its new possibilities incorporating high-performance artificial intelligence for cancer assessment has been expressed in a plethora of validation and research studies and indicates the emerging revolution in pathology that is about to shift the way we treat cancer patients.

However, for broad adoption, a holistic integration of DP into the manifold landscapes of institutions and labs is crucial: crucial to facilitate the clinical workflows for pathologists, instead of adding complexity with more tools and programs, crucial for large-scale research and the development of novel high-performing computational models through the systematic digital access to the vast slide archives in the departments, and crucial for the educational programs to benefit from the concurrent access and download of didactic slides, on-site and remote. With our solution meeting those needs, we hope to inspire other pathologists and to provide useful guidance for their successful digital transformation.

## FUNDING

This research was funded in part through the National Institutes of Health/National Cancer Institute Cancer Center Support Grant P30 CA008748.

## AUTHOR CONTRIBUTIONS

PJS, LG, DVKY, and TJF implemented the digital pathology solution. JS, M-HJ, OA, LC, and AM lead the scanning operation. PJS, MGH, CV, SC, DDG, CSS, LKT, CE, YY, SJS, DK, MH, and VER validated the viewer and HoBBIT workflow. PJS, MGH, ES and JP lead clinical integration. PJS, JSZ and JG led the integration into cBioPortal. PJS, YSK, UKB, and MHAR lead the integration into PPBC. NHGP and JCP lead the integration into the data center and computing cluster. PJS, MM and LG led the usability for education. CV coordinated and lead research projects. All authors contributed to the manuscript.

## SUPPLEMENTARY MATERIAL

Supplementary material is available at J*ournal of the American Medical Informatics Association* online.

## Supplementary Material

ocab085_Supplementary_Data

## DATA SHARING

The software underlying this article was derived from sources in the public domain at https://openslide.org/, http://openseadragon.github.io/ and https://github.com/bgilbert/anonymize-slide.
