# Supplementary material for: Integrated digital pathology at scale: A solution for clinical diagnostics and cancer research at a large academic medical center
Source: J Am Med Inform Assoc. 2021 Jul 14;28(9):1874–84. doi: 10.1093/jamia/ocab085 (PMC8344580; doi:10.1093/jamia/ocab085)
Supplement: ocab085_Supplementary_Data [file ocab085_supplementary_data.docx]

# Supplementary Information

Schüffler et al.: Integrated digital pathology at scale: A solution for clinical diagnostics and cancer research at a large academic medical center


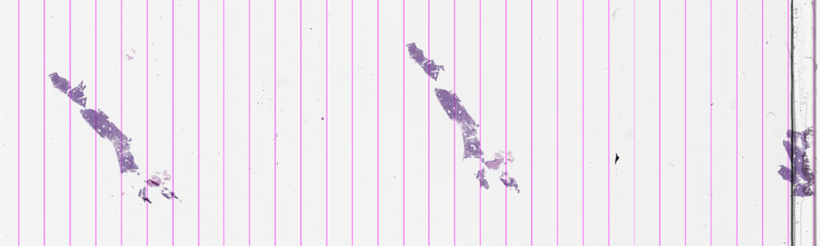

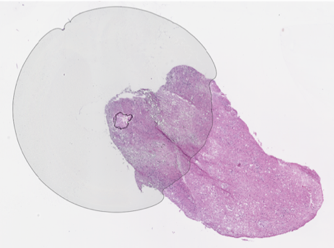

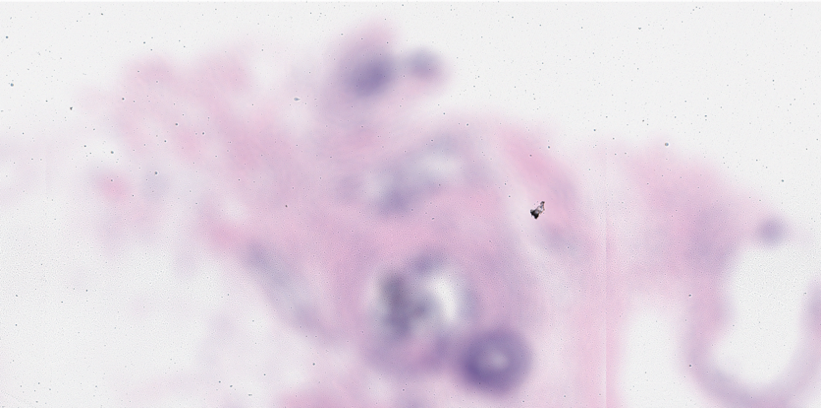

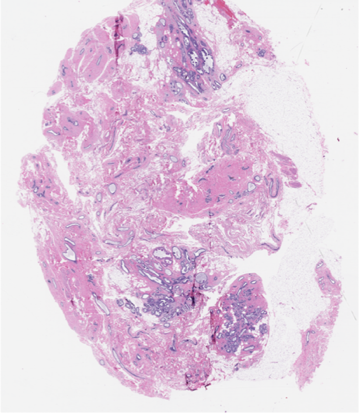


**Figure S 1**: Four examples of scanning artifacts, air bubbles, out-of-focus, and missing tissue, all passing the scanner’s quality control. These artifacts have been discovered and reported by the user via our viewer, such that the slides have been re-scanned.


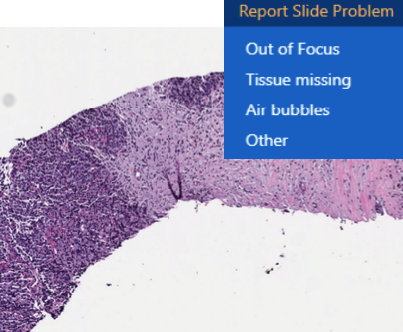


**Figure S 2**: In our system, pathologists can immediately interact in the WSI viewer to report any poor quality of the slide, in case it has been missed by the previous QC steps. A link to the accession number and slide is sent to the scanning team via email with reason of the report, such that the slide can quickly be rescanned.

## Hospital Integration


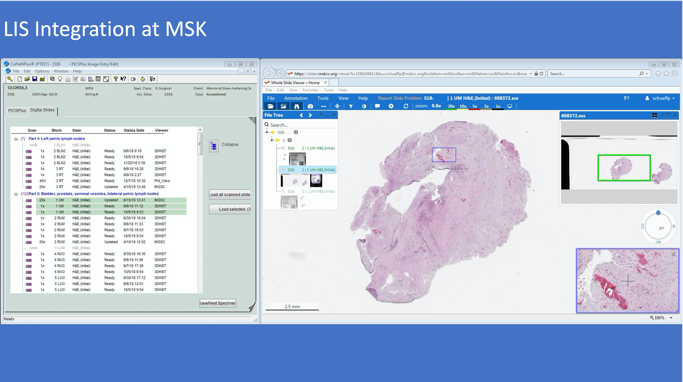

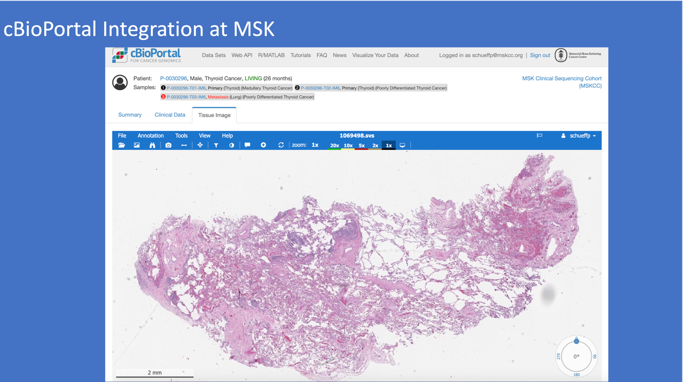


**Figure S 3**: Examples of the connection of the viewer to CoPath (left) and cBioPortal (right). While the CoPath integration serves for clinical purposes and includes the display of PHI, cBioPortal is solely research based and the slides are displayed deidentified.

## HoBBIT implementation

An architecture diagram of HoBBIT is shown in **Figure S 4**. The core of HoBBIT is an on-premises SQL database to store clinical data and pathology reports from the AP-LIS – notably data elements from digitized cases that are useful for research. The HoBBIT database executes a daily pull of all pathology cases containing newly scanned slides with information about the images scanned, tissue descriptions, and diagnosis.

In addition to pathology case data, the HoBBIT database maintains the inventory of image IDs from the AP-LIS and their corresponding file locations. File location information is not available in the AP-LIS, as it is maintained in the separate vendor IMS, and each IMS maintains their own inventory. To rectify this, the HoBBIT system executes distinct image inventory processes (one per vendor, i.e. Leica, Philips, 3DHistech) by which it extracts the file locations and stores them alongside the image IDs in the HoBBIT database. This allows HoBBIT to serve as a universal image file locator across vendor platforms.


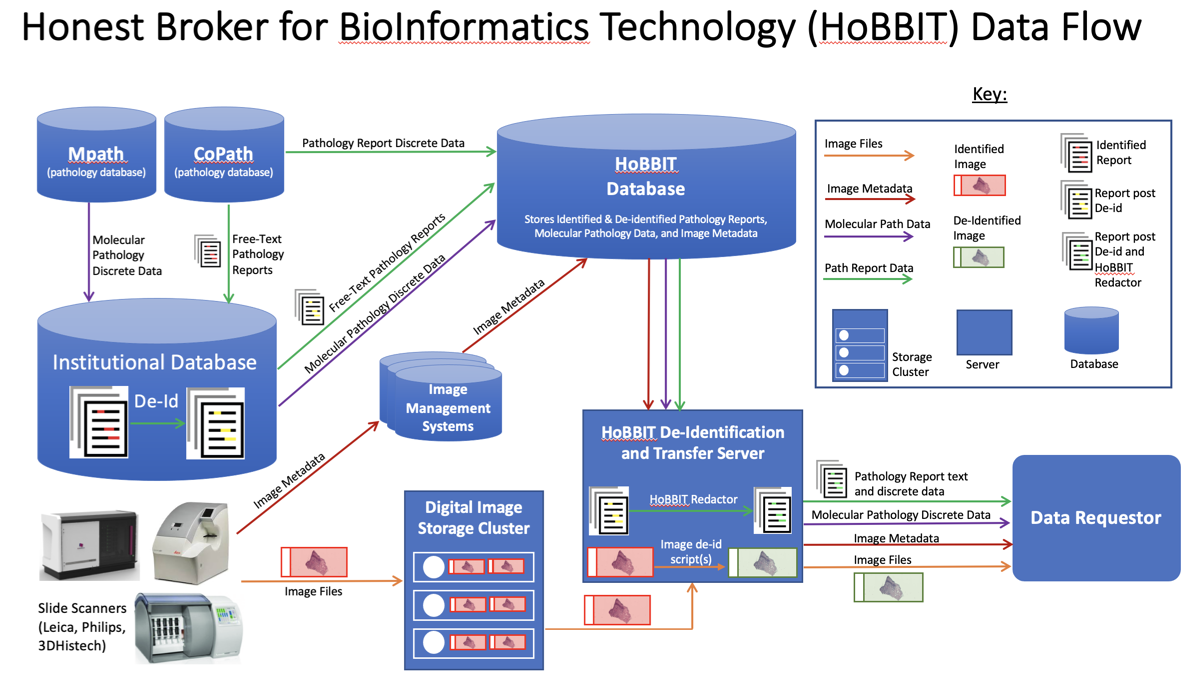


**Figure S 4**: Architecture Diagram of HoBBIT. HoBBIT receives pathology report data and molecular pathology data from the pathology databases of CoPath, MPath and the Institutional Database, WSI meta data from the scanners’ image management systems and image files from the digital image storage cluster. It assembles and de-identifies those data sets and provides them to the data requestor (inside MSK, outside MSK, computer cluster or other).

## Slide Downloads


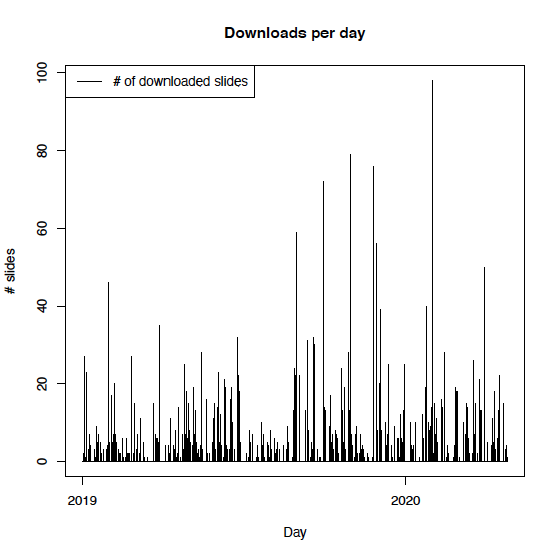


**Figure S 5**: Number of downloads of anonymous slides for educational purposes per day. Download is limited to a maximum of 30 anonymous slides per month and user. In 2019, 2135 anonymous WSIs have been downloaded. Anonymization of slides happens before download, removing the label image, the macro image and specific fields of the slide metadata.


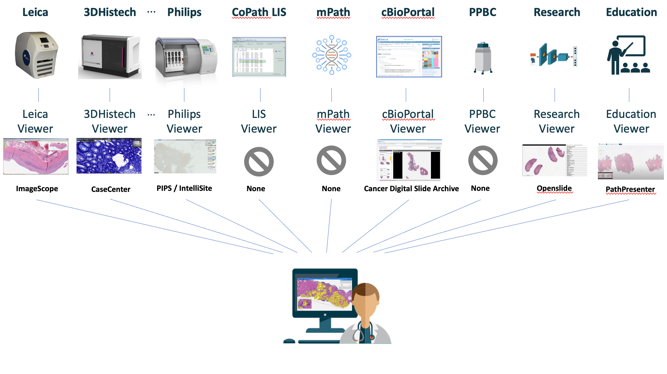

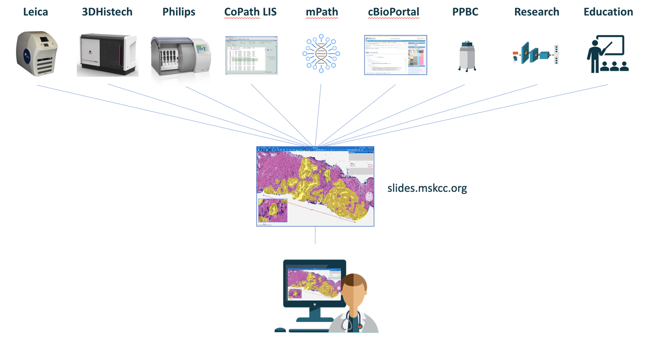


**Figure S 6**: Illustration of the digital platform’s integration in the hospital. Instead of using multiple viewers coming with every system needed in the hospital (left), the web-based viewer can interact with all of them, making it a universal tool for the investigation of DP slides. It is vendor-agnostic and supports file formats of the major scanner vendors.

## Slide viewer implementation

The universal slide viewer is implemented as a C# web application running on a dedicated physical server in our data center. The Windows 2019 server’s specification comprises 2 processors at 2.20GHz, 44 cores, 768GB RAM and 8TB NVMe storage for fast caching, tile serving and annotation. The application uses a local Redis database to store user preferences, system logs and image annotations. To ensure redundancy on a server failure, we maintain a fail-over server with the same specifications as the production server.

The internal architecture uses openslide, [65] an open-source library supporting digital slide formats from Leica, Hamamatsu, Sakura, Trestle and Ventana (Roche). Additionally, we use specific proprietary software development kits (SDKs) from Philips[66] and 3DHistech, [67] in order to read their specific file formats and to build the capability for visualizing z-stacking, making the viewer vendor-agnostic.

On the front-end, the library openseadragon[68] is incorporated to visualize and navigate through parts of the WSI. It supports imaging of multiple layers, such that it can also be used to show annotations such as measurements, labelling overlays, prediction overlays and others. To confirm the correct display of digital slides at different magnification levels across different scanner vendors, we employed a calibration slide with a printed ruler on a micrometer scale (see **Figure S 7**).

To efficiently stream image data from the central storage to the pathologist’s workstation, we follow a dedicated caching mechanism: every WSI opened by a pathologist is completely copied from the storage server to the viewer server. This process needs to be fast enough to copy the gigabyte sized files within seconds, and the viewer server is therefore connected to the storage with two 10 Gb/s connections. From the viewer server, only those subregions of the image that are currently displayed on the pathologist’s screen are streamed to the client. As these are typically few tiles of a few kilobytes each, a standard connection of the pathology workstations to the viewer server of 1 Gb/s is sufficient for a seamless experience.

## Calibration Slide

We use a calibration slide to validate the accuracy of the measurement tool. The glass slide contains a scale in the resolution of 10μm. We scanned that slide with different scanners (Philips UFS, 3DHistech P1000, and Leica AT2 and GT450) to verify that resolution scale bars and measure tools display the correct values. **Figure S 7** shows the calibration slide at a high level.

While Leica's AT2 and GT450 and 3DHistech's P1000 scanners could easily scan the slide, Philips' UFS scanned the slide first unsharp, as the scanner had problems to set correct focus points due to the little and thin content on the slide.


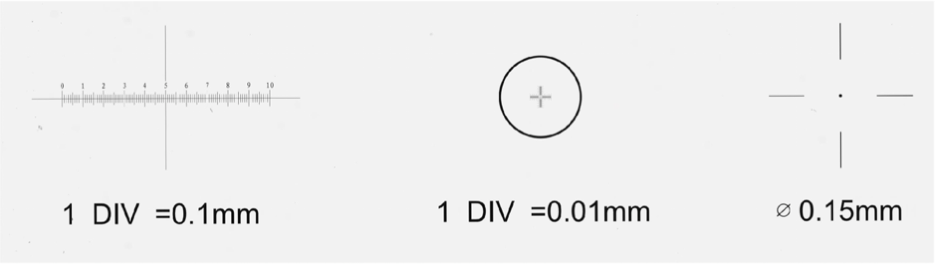


**Figure S 7**: The calibration slide contains scales at a resolution of 0.1 mm and 0.01 mm that can be used to verify scale bars and measurement tools

## Slide Viewer Feedback Studies

In the first feedback study, eight pathologists used the slide viewer to fulfill a list of predefined tasks: Open a case in the viewer, navigate to a particular cancerous slide of the case, identify the cancer and measure the cancer diameter. Then navigate to another case and repeat the same steps. In each step, the user was asked to note if the tasks were easy or hard to fulfill and to note comments. This session was conducted at one timepoint in one room, such that feedback with each pathologist could be exchanged via the script and also in a short discussion with the study leader. A similar study with different cases was conducted a year later with twelve pathologists. The main focus of the two sessions was to reserve dedicated time with developers and pathologists to discuss the present state and future direction of the current system.
